# Evaluation of postoperative feeding strategies in children with intestinal atresia: A single-center retrospective study

**DOI:** 10.3389/fped.2022.953852

**Published:** 2022-09-14

**Authors:** Jiepin Wang, Haozhong Xu, Junxiang Wang, Dong Xiao

**Affiliations:** ^1^Department of General Surgery, Shenzhen Children's Hospital, Shenzhen, China; ^2^Medical College, Shantou University, Shantou, China

**Keywords:** enteral nutrition, post-surgery, prognosis, intestinal atresia, feeding strategy

## Abstract

**Objectives:**

Postoperative enteral nutrition has a significant influence on the prognosis of patients with congenital intestinal atresia. Currently, there is no precise guidance on enteral nutrition management. This study aimed to compare the outcomes of different feeding strategies based on the initial volume and daily advancement in postoperative patients with congenital intestinal atresia.

**Methods:**

This study was a retrospective study collected from October 2019 to July 2021 in Shenzhen Children's Hospital. According to the initial volume and daily advancement, the patients were divided into high-dose group and low-dose group. General basic information such as age, sex, and lesion type was gathered. The postoperative outcome included the levels of hemoglobin (HGB), albumin (ALB), alanine aminotransferase (ALT), aspartate aminotransferase (AST), direct bilirubin (DB), length of stay, length of total PN, time to reach 100% enteral nutrition (EN) (120 kcal·kg-1·d-1), infection incidence and intestinal failure associated liver disease (IFALD) incidence (DB>2 mg·dL-1).

**Results:**

In total, 32 patients with congenital intestinal atresia were identified. There was no significant difference between the high-dose group and the low-dose group in the baseline characteristic. The length of time to reach 100% (*p* = 0.001) enteral nutrition and postoperative hospital stay (*p* = 0.092) were shorter in the high-dose group. In the high-dose group, patients at discharge were with not only lower levels of DB (*p* = 0.009), AST (*p* = 0.109) and ALT (*p* = 0.045) but also higher level of ALB (*p* = 0.459) and hemoglobin (*p* = 0.354). The incidence of IFALD was significantly lower in the high-dose group (*p* = 0.032). There was no significant difference in the overall incidence of postoperative complications.

**Conclusions:**

Within the limitations, the findings of this study suggest that High-dose feeding (initial volume>15 ml·kg-1·d-1, daily advancement>10 ml·kg-1·d-1) is beneficial for the prognosis of patients diagnosed with congenital intestinal atresia treated by intestinal.

## Introduction

Congenital intestinal atresia is one of the common congenital anomalies of the gastrointestinal tract in newborns, including duodenal atresia, small intestine atresia, and colonic atresia. There is about 95% of obstructions in the neonatal period are due to intestinal atresia (1). In Europe, intestinal atresia occurs in about one of every 2,000 newborns, which is a rising trend (2). The newborns diagnosed with congenital intestinal atresia usually require emergency surgery. Feeding difficulties occur in the early postoperative period, which causes the dependence on long-term parenteral nutrition (PN). Therefore, appropriate nutrition management of patients with intestinal atresia after surgery is critical for prognosis.

Compared to PN, enteral nutrition (EN) is recommended as the preferred route for the development of the gastrointestinal system. Common complications of long-term PN include PN-associated liver disease (PNALD) and infection (3, 4). Due to the lack of EN, degeneration of intestinal barrier function and insufficient translocation of bacteria often occur, which stimulates systemic or located inflammation (5–7). However, the application of EN in patients with intestinal atresia after surgery is controversial. Different feeding strategies vary on the timing of first postoperative feeding, initial volume, and daily advancement. In determining which feeding strategy is better, bowel adaptation, fecal production, linear growth, and clinical outcome should be considered.

This study aimed to evaluate the clinical outcomes of different feeding strategies in postoperative patients with congenital intestinal atresia. By comparing two different strategies, we looked forward to finding out the appropriate volume of initial feeding and daily advancement which can be beneficial for prognosis.

## Patients and methods

### Patients

This was a retrospective study approved by Shenzhen Children's Hospital. Patients with congenital intestinal atresia were treated at the neonatal surgery department of Shenzhen Children's Hospital from October 2019 to July 2021. The subjects included patients diagnosed with intestinal atresia. Patients with severe malformations of other systems, extreme malnutrition before surgery, or other preoperative complications (such as necrotizing enterocolitis, gastrointestinal perforation, etc.) were excluded. After clinical stabilization, small bowel resection and anastomosis were performed. The most common technique is resection of proximal dilated and atretic bowel with primary end-to-end anastomosis using 4–0 Vicryl threads. Confirmation of continuity of intestine was checked with the passage of warm saline solution in the distal atretic small bowel. After the enteroplasty, patients were divided into two groups according to the feeding strategies. The application of different feeding strategies was based on not only the surgeon's preference but also the clinical situation of the patients. The conditions of the patients included bloating, fever, and volume of gastrointestinal decompression. The patients were divided into two groups through initial volume based on previous research about feeding guidelines. It is suggested that feeding guideline implementation with higher initial feeding volume was well tolerated (4). In the high-dose group, the patients were given an initial volume of deeply hydrolyzed formula milk > 15 ml·kg-1·d-1 and the rate of advancement was >10 ml·kg-1·d-1. While in the low-dose group, the patients were given an initial volume of deeply hydrolyzed formula milk <15 ml·kg-1·d-1 and the rate of advancement was 0–10 ml·kg-1·d-1. Both groups of patients received similar preoperative interventions including preoperative intravenous antibiotics, bowel preparation, and anesthesia administration. The surgeries were performed by the same team that specialized in intestinal anastomosis.

### Variables

Numerous clinical factors were collected retrospectively in the electronic medical records as follows: sex, age, birth weight, gestational age, diagnosis, the length between atresia and the ligament of Treitz, total resection length, the levels of hemoglobin (HGB), albumin (ALB), alanine aminotransferase (ALT), aspartate aminotransferase (AST), direct bilirubin (DB), length of stay, length of total PN, time to reach 50% EN (60 kcal·kg-1·d-1), time to reach 100% EN (120 kcal·kg-1·d-1), infection incidence and IFAND incidence (DB>2 mg·dL-1). Patients were monitored for postoperatine complications such as an anastomotic leak, vomiting, diarrhea, infection, etc. Every complication was noted carefully.

### Statistical methods

Statistical analysis of data was performed by SPSS version 19.0 at a significance level of *p* < 0.05. For continuous variables, descriptive statistics were calculated and were reported as mean ± SD. Differences in continuous data between the groups were evaluated using the independent sample *t*-test. Categorical variables were described using frequency distributions. Differences in categorical data between the groups were evaluated using a chi-squared test or the Wilcoxon-Mann-Whitney exact test.

## Results

### Baseline characteristics

In total, 32 patients were considered eligible for the study. Seventeen patients were enrolled in the high-dose group and 15 patients were enrolled in the low-dose group. There were no significant differences in the baseline characteristic between the two groups except birth weight (*p* = 0.033). The baseline characteristics are displayed in Table 1.

**Table 1:** Baseline characteristics stratified by postoperative feeding strategies.

		**High-dose group**	**Low-dose group**	***p*-value**
Cases (*n*)	Total	17	15	
	Male	10	5	0.178
	Female	7	10	
Age (h)		19.65 ± 17.83	16.60 ± 13.71	0.596
Gestational age(w)		35.82 ± 2.09	34.60 ± 3.24	0.21
Birth weight(kg)		2.87 ± 0.61	2.35 ± 0.68	0.033
Time to initiate EN(d)		15.26 ± 8.95	13.72 ± 8.48	0.595
Length between atresia and the ligament of Treitz (cm)		67.65 ± 47.73	55.13 ± 39.10	0.428
Length of intestinal resection (cm)		23.82 ± 6.73	22.86 ± 11.38	0.771

### The length of PN, hospital stay, time to reach 50 and 100% EN

In the high-dose group, the length of PN was 19.11 ± 11.28 days, as for the low-dose group, it was 24.06 ± 12.51 days. There was a significant difference (*p* < 0.001) in the length of time to reach 50% EN and 100% EN between the two groups. In the high-dose group, the time to reach 50% EN and 100% EN is significantly shorter. The length of postoperative hospital stay was 38.35 ± 20.68 days for the high-dose group and 49.86 ± 16.12 days for the low-dose group with a borderline level of statistical significance (*p* = 0.092).

### Biochemical indicators and IFALD incidence

The levels of evaluating factors including DB, AST, ALT, ALB, and HGB were recorded before and after EN. There is no statistically significant difference in the levels of DB, AST, ALT, ALB, and HGB before EN between the two groups. After the implementation of different feeding strategies, the evaluating factors performed better in the high-dose group. In the high-dose group, the levels of DB (*p* = 0.009), AST (*p* = 0.109), and ALT (*p* = 0.045) were found to be lower, meanwhile the levels of ALB (*p* = 0.459) and HGB (*p* = 0.354) were higher. In addition, the level changes of DB before and after feeding in the high-dose group were significantly higher than that in the low-dose group, which was statistically significant. The IFALD incidence was 5(17) in the high-dose group and 11(15) in the low-dose group with a significant difference (*p* = 0.032). In the high-dose group, postoperative vomiting developed in two patients. As for the low-dose group, postoperative vomiting developed in two patients, and one appendicitis was reported. No significant differences were found in the incidence of infection and postoperative complications in the high-dose group and the low-dose group. The postoperative outcomes are displayed in Table 2. The weight gain during hospitalization is displayed in Table 3. Compared with birth weight, both the high-dose group (*p* = 0.003) and low-dose group (*p* < 0.001) had significantly higher weights at discharge.

**Table 2:** Comparisons of postoperative outcome in children with intestinal atresia stratified by postoperative feeding strategies.

		**High-dose group**	**Low-dose group**	***p*-value**
Cases (*n*)		17	15	
DB (μmol/L)	Pre-feeding	16.06 ± 10.64	17.19 ± 9.84	0.759
	Post-feeding	11.75 ± 5.91	21.54 ± 12.94	0.009
	change	−4.37 ± 9.71	4.34 ± 12.61	0.035
AST (IU/L)	Pre-feeding	19.23 ± 6.43	27.46 ± 19.65	0.113
	Post-feeding	23.05 ± 8.72	31.20 ± 18.11	0.109
	change	4.64 ± 7.56	3.73 ± 26.17	0.891
ALT (IU/L)	Pre-feeding	8.94 ± 4.05	12.33 ± 11.41	0.262
	Post-feeding	10.64 ± 4.12	24.06 ± 26.13	0.045
	change	1.70 ± 4.39	11.73 ± 25.42	0.119
ALB (g/L)	Pre-feeding	32.62 ± 3.59	31.18 ± 4.93	0.346
	Post-feeding	33.77 ± 3.69	32.81 ± 3.54	0.459
	change	1.14 ± 4.74	1.63 ± 4.86	0.777
HGB (g/L)	Pre-feeding	121.76 ± 27.49	111.60 ± 38.44	0.392
	Post-feeding	102.94 ± 27.49	96.13 ± 19.38	0.354
	change	−18.23 ± 18.94	−15.46 ± 37.25	0.746
Hospital stay (d)	38.35 ± 20.68	49.86 ± 16.12	0.092
50% EN(d)	5.47 ± 1.94	9.80 ± 3.87	<0.001
100% EN(d)	9.58 ± 3.16	19.13 ± 10.16	0.001
Discharge weight (kg)	3.27 ± 0.38	3.02 ± 0.62	0.030
Length of PN(d)	19.11 ± 11.28	24.06 ± 12.51	0.249
IFALD (*n*)	5	11	0.032
Infection (*n*)	1	2	0.589
Complication (*n*)	2	3	0.645

DB, direct bilirubin; AST, aspartate aminotransferase; ALT, alanine aminotransferase; ALB, hemoglobin; HGB, hemoglobin; EN, enteral nutrition; PN: parenteral nutrition.

**Table 3:** Comparison of weight change stratified by postoperative feeding strategies.

	**Birth weight (kg)**	**Discharge weight (kg)**	**Weight change (kg)**	***p*-value**
High-dose group	2.87 ± 0.61	3.27 ± 0.38	0.33 ± 0.44	0.003
Low-dose group	3.02 ± 0.62	2.35 ± 0.68	0.66 ± 0.34	<0.001

## Discussion

Postoperative EN of congenital intestinal atresia has received more and more attention in recent years. However, there is no guideline to state the suitable feeding strategy. The traditional idea is that feeding should be initiated when the patient has defecated and the normal bowel function has recovered. PN plays a role in providing nutritional support to the patients during the fasting period. However, some studies have pointed out that long-term PN can cause complications such as intestinal mucosal atrophy, cholestatic liver disease, and infection (4). Therefore, the current mainstream of studies about postoperative nutrition management is aimed to establish an early feeding guideline, which can meet the nutritional requirements of life maintenance and development for patients with intestinal surgery. DR Shores et al. compared the prognosis of infants with intestinal surgery after implementing a new feeding guideline and concluded that higher initial feeding volume can not only shorten the time to reach 50% of goal EN calories but also reduce the incidence of PN-related liver disease (4). Previously, our team performed a randomized controlled trial which concluded that early feeding can shorten the length of postoperative hospital stay without an increase in postoperative complications in patients with congenital duodenal atresia (8). However, there is no theoretical guidance with high reliability of the initial feeding volume and daily advancement.

After the surgical resection of the obstructed intestinal segment, the remaining segment undergoes a series of physiological changes. First of all, the stress stage of the intestines leads to the dysfunction of intestinal peristalsis. The incidence of postoperative diarrhea increases, which leads to electrolyte imbalance and dehydration (9). After PN and fluid therapy, the clinical condition of the patients entered a stable period, during which the incidence of liver disease increased greatly. As is reported, liver failure is an important cause of postoperative death in infants (9). In this study, compared to the low-dose group (initial volume<15 ml·kg-1·d-1, daily advancement <10 ml·kg-1·d-1), the risk of IFALD after feeding was lower in patients in the high-dose group (initial volume >15 ml·kg-1·d-1, daily advancement >10 ml·kg-1·d-1). The levels of DB, AST, and ALT were significantly lower in the high-dose group. Elevated liver function indexes such as direct bilirubin, liver enzymes, and alkaline phosphatase are important manifestations of cholestasis caused by IFLAD (10). It can be seen that high-dose feeding has a benign effect on the recovery of postoperative liver function in patients with congenital intestinal atresia.

Postoperative EN should process step by step. As the clinical condition is suitable for EN, feeding should be started as soon as possible. DR Shores et al. pointed out that higher initial feeding volume was well tolerated and resulted in the faster achievement of 50% goal EN calories (4). Perks et al. recommended continuous pumping for 24 h at first and then switching to intermittent feeding as the child adapts. The authors also recommend setting the feeding rate to 10–35 mL/kg/d (11). Some scholars have carried out related studies and the results have shown that there is no significant difference in the risk of NEC caused by continuous feeding and intermittent feeding in low-weight children. According to the results of our study, the length of hospital stay and time to reach 50% goal EN calories was shorter in the high-dose group, which was consistent with the previous studies.

The limitations of this study are the small sample size and single-center. Although these limitations can't be ignored, this study reflects the experience of EN in patients with relatively rare surgical disorders. We plan to draft a feeding guideline and conduct a randomized controlled trial to evaluate the guideline.

## Conclusion

Higher initial feeding volume and faster daily advancement after surgery for patients with congenital intestinal atresia are well-tolerated and effective for the decrease of hospital stay and time to reach 100% of goal EN calories. We also reported improved liver function in the high-dose group.

## Data availability statement

The raw data supporting the conclusions of this article will be made available by the authors, without undue reservation.

## Ethics statement

Ethical review and approval was not required for the study on human participants in accordance with the Local Legislation and Institutional Requirements. Written informed consent to participate in this study was provided by the participants' legal guardian/next of kin.

## Author contributions

DX: conceptualization, supervision, and funding acquisition. JiW: methodology, investigation, data curation, and writing-original draft. JuW and HX: writing-review and editing and validation. All authors contributed to the article and approved the submitted version.

## Funding

Science and Technology Program of Shenzhen, China (General Program: JCYJ20210324135409025).

## Conflict of interest

The authors declare that the research was conducted in the absence of any commercial or financial relationships that could be construed as a potential conflict of interest.

## Publisher's note

All claims expressed in this article are solely those of the authors and do not necessarily represent those of their affiliated organizations, or those of the publisher, the editors and the reviewers. Any product that may be evaluated in this article, or claim that may be made by its manufacturer, is not guaranteed or endorsed by the publisher.
